# Loot box spending is associated with greater distress when normalized to disposable income: a reanalysis and extension of Etchells *et al*. and Xiao *et al*.

**DOI:** 10.1098/rsos.231264

**Published:** 2025-07-16

**Authors:** Aaron Drummond, Lauren Camille Hall, Emily Lowe-Calverley, Eamon Garrett, James D. Sauer

**Affiliations:** ^1^ School of Psychological Sciences, University of Tasmania, Hobart, Tasmania, Australia; ^2^ University of Tasmania, Hobart, Australia

**Keywords:** loot boxes, gambling, gaming, video games, microtransactions, distress

## Abstract

Loot boxes are purchasable, randomized rewards available in some video games. These mechanisms share important psychological and legal similarities with conventional forms of gambling. While research has consistently demonstrated an association between risk of gambling harm (as indexed by problem gambling symptomatology) and spending on loot boxes, findings are mixed on whether purchasing loot boxes is associated with psychological distress. Divergent findings may reflect uncontrolled socio-economic confounds. We reanalyse two recent, publicly available datasets for associations between loot box spending and psychological well-being and distress. We find divergent results. In one study, when loot box spending is normalized to participants’ disposable income, loot box spending is associated with psychological distress, translating to increased risk of extreme distress, partially or fully accounted for by Problem Gambling Risk Status. In contrast, the other study, using a shorter distress scale, showed no relationships between loot box spending and distress irrespective of how spending was considered. Our findings suggest that loot box spending should be considered in the context of available financial means, albeit with improved measures. Our work concords with work on conventional gambling showing some gamblers bet more than they can afford to lose, meeting quintessential definitions of financial harm.

## 1. Introduction

Loot boxes—purchasable, randomized rewards available in some video games—share psychological and legal similarities with conventional forms of gambling [1,2]. These features are underpinned by the same structural and psychological characteristics as conventional gambling, delivering randomized prizes of varying value on variable ratio reward schedules to players, often in exchange for real-world money [1]. Where these features involve real-world money, they appear to also satisfy most, if not all, of the legal criteria for conventional gambling [2]. Concordantly, there is a robust association between loot box spending and problem gambling symptomatology, with individuals at greater risk of gambling harm consistently spending more on these mechanisms than peers at lower risk of gambling harm [3]. Moreover, this spending pattern is consistent across income brackets such that, within income brackets, those at highest risk of gambling harm spend more than those at lower risk [4,5]. In fact, Garrett *et al.* [4] demonstrated that those at greatest risk of gambling harm often spent more on loot boxes than those at low risk even when those at lower risk were in higher income brackets.

Presently, it remains unclear whether problem gambling symptoms contribute to, or are exacerbated by, loot box engagement. Either possibility is potentially problematic [6–8]. Research suggests that removing loot boxes from a game results in a reduction in spending specifically for individuals at greatest risk of gambling harms [9], and emerging evidence suggests that loot box engagement predicts migration to conventional forms of gambling [10–12]. However, it remains unclear whether this migratory pathway is the direct result of loot box engagement or pre-existing personality characteristics which render some individuals more vulnerable to both loot box spending and gambling behaviours, or if indeed loot boxes act as a gateway to the development of future gambling activity.

Nonetheless, even if loot boxes simply appeal more to individuals at increased risk of gambling harm, this would imply increase, and disproportionate, revenue is being derived from a vulnerable population at risk of gambling harms [6]. Data consistently suggest substantially positively skewed spending distributions, sometimes with players reporting spending over US$1000 per month on loot boxes [6]. Furthermore, increased spending on loot boxes is associated with increased post-purchase consumer regret: players who spend more on loot boxes regret their purchases more than those who spend less on these mechanisms [13]. Taken together, these findings suggest that for at least some players (especially players with problem gambling symptoms and limited income), loot boxes might pose risks of financial harm.

A related issue is whether loot box spending is associated with increased psychological distress (e.g. poorer psychological well-being, and increased risk of anxiety and depression). Recent papers present divergent evidence on this issue. Drummond *et al.* [6] demonstrated a small positive association between psychological distress and loot box spending, a result replicated in a later article by Hall *et al.* [14]. When grouped into risk categories, reanalysis of these data suggested that individuals who purchased loot boxes were at an 87% higher risk of also experiencing extreme psychological distress compared to individuals whom did not purchase loot boxes [15]. Interestingly, this finding was only partially explained by problem gambling symptoms: when problem gambling symptoms were statistically controlled for, individuals who purchased loot boxes (compared to those who did not) remained 27% more likely to experience extreme psychological distress. In contrast however, Etchells *et al.* [16] found that, while associated with problem gambling symptomatology, loot box spending was unrelated to psychological distress. Further, in a study examining loot box spending in a sample of Chinese residents, Xiao *et al.* [17] reported a negative association between loot box spending and psychological distress. Thus, whether loot box spending is associated with psychological distress, and the precise nature of this relationship, remains unclear.

Individual difference factors may explain why these results diverge. Participants’ country of residence is one such factor. Drummond *et al*.’s [15] participants all resided in societies with average annual incomes above the mean for the OECD [18]. In contrast, Xiao *et al*.’s [17] sample was drawn from China where the average annual income is well below the mean for the OECD [18], and almost half of Etchells *et al*.’s sample [16] comes from countries below the average annual income for the OECD. Lower income has consistently been associated with greater psychological distress [19–21]. Thus, countries which typically have lower socio-economic status and household income may also be expected to have increased psychological distress, and this confounding factor may overshadow any impact that loot box purchasing has upon psychological distress. Thus, it is important to control for the availability of financial resources in some way to better understand the association between loot box spending and psychological distress.

Fortunately, Etchells *et al.* [16] had the foresight to collect data regarding not only participants’ income, but also participants’ *disposable* income, and Xiao *et al*. [17] faithfully replicated this method. Of all forms of income, disposable income is likely to be the most relevant to psychological distress as it indexes an individuals’ ability to engage in discretionary spending above those mandatory living expenses. As loot boxes are a discretionary purchase this metric appears highly relevant in our understanding of the association between loot box spending and psychological distress. Specifically, in this case, data on individuals’ disposable income allow us to test whether individuals who spend a higher proportion of their disposable income on loot boxes might experience greater psychological distress. Here, we employed analysis methods undertaken in recent research on the issue [15] to reanalyse Etchells *et al*.’s [16] and Xiao *et al*.’s [17] data to understand whether individuals who spend more on loot boxes as a proportion of their disposable income are also more likely to experience negative psychological (as well as financial) outcomes. We hypothesized that individuals who spent a greater proportion of their disposable income on loot boxes would be at greater risk of suffering from extreme psychological distress compared to peers who spent less of their disposable income on the mechanism.

## 2. Method

We undertook a Bayesian reanalysis of Etchells *et al*.’s [16] and Xiao *et al*.’s [17] publicly available datasets, which contain data on loot box spending and psychological distress. These studies collected a range of data on loot box engagement and spending, psychological distress and financial indicators. Data in Etchells *et al*.’s [16] dataset were converted by the researchers to a UK currency using the exchange rates on the 21 May 2021. Data in Xiao *et al*.’s [17] dataset were all reported in Chinese Yuan and did not require currency conversion. Here, we used collected metrics of loot box spending, disposable income and psychological distress (via the Kessler-10 (K-10) psychological distress inventory), to estimate the relative risk of a participant experiencing extreme psychological distress in a binomial logistic regression analysis. All analyses were conducted in JAMOVI version 2.3.28.

One facet of the data that is worth discussing is the choice of variables employed in our analysis. In addition to collecting psychological distress, Etchells *et al.* [16] collected data measuring psychological well-being using the Warwick–Edinburgh Mental Wellbeing Scale (WEMWBS) [22]. This scale captures various facets of well-being, but its purpose differs markedly from the K-10 [23]. The K-10 seeks to capture the frequency of feelings associated with psychological disorders such as anxiety and depressive disorders, and is highly predictive of the presence of such disorders [23]. In contrast, the WEMWBS is not a clinically predictive scale, being designed to capture, in short form, positive aspects of a broad conceptualization of psychological well-being, including positive affective, positive cognitions and positive physiological dimensions of well-being [24]. Concordantly, the focus on positivity shows only moderate correlations with clinical scales of depression and anxiety [24]. Evidence further suggests that the WEMWBS may not perform well at clinically low levels of well-being, suggesting it may be inappropriate for detecting the presence of psychological disorders such as anxiety and depression [24]. It is also important to note that the concept of psychological well-being is not antithetical to the presence of psychological distress, and evidence suggests that they play complementary roles in psychological well-being [25]. In other words, while both scales are useful to understand the complete picture of psychological well-being and distress, the WEMWBS only indexes positive aspects of mental health, while the K-10 only indexes markers of distress. Both scales have been designed to be adaptable for cross-cultural settings to allow for national and international indexing and comparison. The WEMWBS also does not contain any categorical scoring for those at risk of anxiety and depression. As we are most interested in the risk of adverse psychological outcomes associated with engagement with loot boxes here, we therefore focus upon the K-10 scale, but provide supplementary analysis of the WEMWBS for additional context.

We normalized Etchells *et al*.’s [16] data to disposable income by dividing each participant’s reported loot box spending by their disposable income. Where both spending and disposable income were 0, a missing case was retained. Where a value for loot boxes was given but disposable income was 0, no ratio was computed. Evidence suggests that inclusion of a ratio variable of this nature is appropriate provided that the raw variables which make up the ratio are also included in the analysis [26]. We believe that within the Etchells *et al.* dataset, disposable income appeared to be the most appropriate financial measure to employ because there appeared no other method to easily account for the widespread differences in cost-of-living present within Etchells *et al*.’s data [16]. As noted earlier, a large proportion of the original dataset were drawn from countries with large income disparities, and thus standardization for cost of living appeared to us to be more necessary than with data drawn from countries with more homogeneity in the cost of living. Monthly incomes were also collected within this project; however, because of disparity in wages across countries in terms of what is considered wealthy or poor, we focussed upon the indices of disposable income to account for those cost-of-living differences across countries.

For the reanalysis of Xiao *et al*.’s [17] data, we employed the same techniques as specified above. However, Xiao *et al*.’s [17] study collected the shorter Kessler-6 (K-6) measure for psychological distress. Scores on Kessler psychological distress scales are often extremely positively skewed, with evidence suggesting that more than two-thirds of participants typically score within the lowest four points on a 40-point scale, with almost 90% of participants scoring in the bottom quarter of the scale’s range [27]. These floor effects and relative invariance of the measure mean that for the vast majority of the sub-clinical population, it is difficult to adequately distinguish subtle variations in psychological distress. Moreover, such sub-clinical variability does not match the intended purpose of the tool. The Kessler tools were designed as screening tools to maximize sensitivity and specificity for detecting those individuals most at risk of a bona fide psychological disorder (i.e. the most distressed decile of the population) as compared to those people who are unlikely to suffer from a mental health disorder [27]. Thus, these tools are ideal, when scores are categorized into severe mental health risks according to the repeatedly validated scoring criteria of the instruments (and their stated purpose for use as a screening tool), for identifying with a high degree of precision, the risk of individuals being severely distressed (i.e. experiencing harms). Loot box research has drawn criticism for failing to adequately demonstrate that engaging with loot boxes is associated with serious harms [28], so the ability to determine whether severe psychological distress typical of bona fide mental health disorders more frequently co-occurs with higher loot box spending is both a timely and important advance. Thus, in both studies, in addition to reanalysing the data as a continuous measure, we recoded the K-10 scale to a dichotomous measure of whether an individual exceeded the recommended cutoffs indicative of extreme psychological distress. This resulted in a cutoff of 30 [23] for Etchells *et al*.’s [16] dataset, and a cutoff of 13 for the K-6 [29] for Xiao *et al*.’s [17] dataset.

## 3. Results

*Etchell et al.’s* [*16*] *data correlational reanalysis*. We employed Etchells *et al*.’s [16] specified beta width of 0.0213 for our analyses. When standardized loot box spending was analysed in place of unstandardized loot box spending, the resulting Bayes factors were dramatically altered. For unstandardized loot box spending, we were able to perfectly replicate Etchells *et al*.’s [16] analyses, revealing moderate evidence in favour of the null for an association between loot box spending and psychological distress (BF_0+_ = 3.25/BF_+0_ = 0.31). For further analysis, we excluded any participant who indicated a negative value of disposable income on the recommendation of a reviewer. In contrast to the above analysis, when including loot box spending, disposable income and loot box spending *as a proportion of disposable income* (LS : DI) in a Bayesian linear regression, analysis revealed extreme evidence that the best model included the main effects of disposable income (*B* = −0.002) and LS : DI (*B* = 8.181) on psychological distress, BF_10_ = 6.11 × 10^25^, *r*^2^ = 0.049. There was extreme evidence that this model was also preferred to a model which only included disposable income (and not LB : DI) by a factor of 169 722. When we excluded participants for whom the financial impact of loot box spending is greatest (i.e. their LS : DI was 0.5 or more), there remained strong evidence (i.e. BF_10_ = 13.35) and a similar effect size (*B* = 7.985) for the association between LS : DI and psychological distress. This suggests two important points—first that a small sub-population of individuals who spend more of their disposable income on loot boxes are at particular risk of also having high levels of psychological distress; and second, that even when these individuals are excluded from the analysis, there remains strong evidence for an association between LS : DI and distress among those spending less than 50% of their disposable income on loot boxes. Thus, when loot box spending was normalized to disposable income, our findings clearly contradict Etchells *et al*.’s [16] claim that there was no association between loot box spending and psychological distress. When this analysis was repeated on the WEMWBS scale, we again found evidence that the best model included main effects of disposable income (*B* = 0.002) and LB : DI (*B* = −5.35), BF_10_ = 4.98 × 10^11^, *r*^2^ = 0.025. There was moderate evidence that this model was preferred over one including only disposable income as a main effect alone, BF_10_ = 4.26, *τ*_b_ = −0.02, BF_0+_ = 11.5. Taken together, this suggests that loot box spending as a function of disposable income is associated with, on average, an 8-point increase in psychological distress as loot box spending reaches parity with disposable income (i.e. 1.0), and a 5-point lower well-being score. Given that differences between mild, moderate and severe mental health categories on the K-10 are only 5 points, an 8-point average change in distress scores is likely to be clinically meaningful, and the *r*^2^ scores indicate small-to-moderate effect sizes.

*Xiao et al.’s* [*17*] *data correlational reanalysis.* For Xiao *et al*.’s [17] dataset, one participant indicated a negative value for disposable income which implied they were spending negative 100% of their disposable income on loot boxes. To correct this, we excluded any person who indicated a negative value of disposable income. We first verified the analysis reported by Xiao *et al.* [17] showing that there was no raw association between loot box spending and psychological distress, *r*_s_ = −0.04, *p* = 0.07. Unlike the Etchells *et al.* [16] reanalysis presented above, the outcome was qualitatively stable when we considered loot box spending as a proportion of disposable income, alongside loot box spending and disposable income, in a Bayesian regression analysis. Analysis suggested that the best model was one incorporating only disposable income, *B* = −0.0001, BF_10_ = 13 548. This model was preferred to one including LB : DI by a factor of 7.66. The same analysis using the WEMWBS scale showed that a model including only disposable income was preferred, *B* = 0.0001, BF_10_ = 8.47 × 10^8^. This model was preferred to a model including LB : DI by a factor of 12.1. Together these data suggest that in Xiao *et al*.’s [17] dataset, there is no association between loot box spending as a function of disposable income and psychological well-being or distress.

*Etchells et al.’s* [*16*] *data binomial regression reanalysis*. Given that Kendall’s Tau-b (*τ*_b_) underestimates effect size relative to Pearson’s *r* [30], and the use of the K-10 as a clinical screening tool and the preponderance of low scores on the K-10 [15,27], linear analyses may be sub-optimal here. Thus, adopting Drummond *et al*.’s [15] method we used a binomial logistic regression to estimate the relative risk of exceeding an extreme distress score of 30+ on the K-10 measure (as is appropriate for this clinical screening tool) [23]. Using Binomial regression analyses, we estimated the odds ratio (ORs) of exceeding the cutoffs for extreme distress as LB : DI increased from 0 to 1.00. As ORs are often unintuitive and frequently incorrectly interpreted as the relative risk to groups (rather than the relative odds for groups) we converted the ORs to relative risk (RR) estimates using Grant’s [31] formula, using the baseline prevalence rate of exceeding the K-10 cutoff for extreme distress of 26.55% in those who spent no money on loot boxes (i.e. unexposed). When included as a continuous predictor with age, gender, loot box spending and disposable income, the relative risk of being extremely psychologically distressed for individuals who spent 100% of their disposable income on loot boxes was twice that of peers who spent 0% of their disposable income on loot boxes, RR = 2.02, *p* = 0.009. Thus, as participants’ spending approaches parity with their disposable income, their risk of experiencing severe psychological distress more than doubles. Although this may seem at face value an implausible amount of spending, the data do not appear to bear this out, with a small tail of participants reporting spending high amounts relative to their disposable income. Approximately 1.4% of Etchells *et al*.’s [16] participants reported spending at least half of their reported disposable income on loot boxes, and approximately 0.3% reported spending 100% or *more* of their disposable income on loot boxes. The full model is presented in table 1.

**Table 1.** Statistical associations between age, gender, loot box spending, disposable income and loot box spending as a function of disposable income, on risk of extreme distress.

predictor	estimate	s.e.	*p*	**odds ratio**	lower	upper
intercept	−0.41563	0.15855	0.009	0.660	0.484	0.900
age	−0.01963	0.00575	<0.001	0.981	0.970	0.992
gender						
female–male	0.47939	0.09894	<0.001	1.615	1.330	1.961
other–male	0.92208	0.37152	0.013	2.515	1.214	5.208
loot box spending	0.00318	0.00253	0.208	1.003	0.998	1.008
disposable income	−5.55 × 10^−4^	1.13 × 10^−4^	<0.001	0.999	0.999	1.000
LB : DI	1.16703	0.44925	0.009	3.212	1.332	7.749

Interestingly, like Drummond *et al*.’s [15] work, including problem gambling status as a categorical factor in this model reduced the relative risk somewhat, RR = 1.67, *p* = 0.083 (see table 2 for the full model). However, unlike Drummond *et al*.’s [15] work, the effect became non-significant, suggesting that the effect of spending on extreme distress is either partially or wholly explained by problem gambler status. This is made more complex by the fact that although we now know loot box purchasers migrate into gambling activities at higher rates [10,11], the role played by loot box engagement in the development of problematic gambling behaviours remains unknown. Note that as would be expected, females, other genders, lower disposable income and moderate or high-risk problem gambling severity index (PGSI) status were associated with significant increases in risk of extreme distress.

**Table 2.** Statistical associations between age, gender, loot box spending, disposable income, loot box spending as a function of disposable income and PGSI status on risk of extreme distress.

predictor	estimate	s.e.	*p*	**odds ratio**	lower	upper
intercept	−0.6367	0.16872	<0.001	0.529	0.380	0.736
age	−0.0189	0.00583	0.001	0.981	0.970	0.993
gender						
female–male	0.5424	0.10090	<0.001	1.720	1.411	2.096
other–male	1.0298	0.37317	0.006	2.801	1.348	5.820
loot box spend	4.32 × 10^−4^	0.00262	0.869	1.000	0.995	1.006
disposable income	−5.48 × 10^−4^	1.15 × 10^−4^	<0.001	0.999	0.999	1.000
problem gambling risk category						
low–none	0.1586	0.11326	0.161	1.172	0.939	1.463
moderate–none	0.5480	0.12227	<0.001	1.730	1.361	2.198
high–none	1.5791	0.21499	<0.001	4.850	3.183	7.392
LB : DI	0.7976	0.46037	0.083	2.220	0.901	5.473

*Xiao et al.’s* [*17*] *data binomial regression reanalysis.* Adopting the same binomial method described above, we reanalysed Xiao *et al*.’s [17] data. Whereas Etchells *et al.* [16] employed the Kessler-10, Xiao *et al.* [17] employed the shorter Kessler-6. Thus, we employed the K-6 criteria of a score of 13 or above to identify individuals who were severely distressed. Analysis showed 10.9% of people who did not buy loot boxes were exceeding the extreme distress cutoff on the K-6, which we used to convert ORs to RRs. When included as a continuous predictor with age, gender, loot box spending, disposable income and PGSI category as controls, the relative risk of being extremely psychologically distressed for individuals who spent 100% of their disposable income on loot boxes was not significantly elevated compared to peers who spent 0% of their disposable income on loot boxes, RR = 1.09, *p* = 0.087. Unlike the previous analysis, excluding PGSI as a predictor did not categorically alter this result. However, it is interesting to note that the risk of extreme distress was also unaffected by age (*p* = 0.39), gender (*p* = 0.46/0.82), disposable income (*p* = 0.16), low-risk gambler status (*p* = 0.19), or moderate-risk gambler status (*p* = 0.96). Full results are presented in table 3.

**Table 3.** Statistical associations between age, gender, loot box spending, disposable income, loot box spending as a function of disposable income and PGSI status on risk of extreme distress.

predictor	estimate	s.e.	*p*	**odds ratio**	lower 95% **CI**	upper 95% **CI**
intercept	−1.6894	0.4480	<0.001	0.185	0.0767	0.444
age	−0.0101	0.0117	0.391	0.990	0.9674	1.013
gender						
male – female	−0.1047	0.1422	0.461	0.901	0.6815	1.190
other – female	0.2640	1.1427	0.817	1.302	0.1387	12.228
disposable income	−3.58 × 10^−5^	2.55 × 10^−5^	0.161	1.000	0.9999	1.000
loot box spending	−9.55 × 10^−5^	7.39 × 10^−5^	0.196	1.000	0.9998	1.000
problem gambling risk category						
low–none	−0.5534	0.4184	0.186	0.575	0.2532	1.305
moderate–none	0.0233	0.4463	0.958	1.024	0.4268	2.455
high–none	1.0295	0.3842	0.007	2.800	1.3186	5.945
missing–none	0.0889	0.3458	0.797	1.093	0.5549	2.153
LB : DI	0.0912	0.0533	0.087	1.096	0.9869	1.216

## 4. Discussion

We reanalysed public data from both Etchells *et al*.’s [16] multinational survey and Xiao *et al*.’s [17] Chinese survey of loot box spending, with a focus on clarifying the discrepancy in findings between these studies and other studies [6,14,15], of the relationship between loot box spending and psychological distress. Three key findings emerged from our reanalysis. First, when considering psychological distress as a continuous measure, loot box spending was associated with increased psychological distress when normalized to disposable income in Etchells *et al*.’s [16] dataset. This was not true for Xiao *et al*.’s [17] dataset. It is unclear why this discrepancy occurs. We speculate it may be due to sociocultural factors, or the restricted range caused by Xiao *et al*.’s using a 6-item rather than 10-item index of psychological distress (we return to these issues below). Second, these findings were mirrored with the measure of psychological well-being (WEMWBS). Etchells *et al*.’s [16] dataset shows greater spending as a function of disposable income is associated with reduced well-being. No such finding occurs in Xiao *et al*.’s [17] dataset.

Third, when psychological distress was considered as a categorical outcome (in keeping with the explicit coding instructions of the K-6 and K-10 scales), for Etchells *et al*.’s [16] dataset players who purchased loot boxes were at elevated risk of experiencing extreme psychological distress as their spending approached parity with their disposable income. This risk was partially or fully mitigated by problem gambler status. Since recent evidence suggests that engaging with loot boxes might act as a migratory pathway to conventional gambling [10,11], it is unclear whether this should be taken to imply that the risk is due to gambling risk more broadly, or whether engaging with loot boxes may lead to both outcomes, causing complex collinearity between the two. Given Brooks & Clark’s [10] research showing a complex bidirectional pathway, we suspect the latter is likely, but further research is needed to clarify these findings.

In contrast, Xiao *et al*.’s [17] dataset did not show any increase in the risk of extreme distress as a function of loot box spending, whether standardized to disposable income or not. It is unclear why this discrepancy occurs. However, it is noteworthy that the K-6 appeared to do a poorer job of identifying extreme psychological distress in this dataset. Many of the conventional predictors of extreme distress, such as being gender diverse, having moderate gambling risk or having poorer disposable income were not associated with elevated risk. Moreover, overall rates of extreme distress were much lower in the Xiao *et al.* [17] data than in Etchells *et al*.’s [16] data. This is further complicated by the potential for sociocultural factors between the People’s Republic of China and the diverse range of predominantly Western countries in Etchell *et al*.’s [16] data. For now, these data lead us to recommend two important directions for the field. First, where possible retaining the full K-10 scale appears appropriate given the lack of discrimination seen by the K-6 in this reanalysis. Further studies should examine the appropriateness of these scales in culturally diverse contexts to better understand when and how they are most appropriately used. Second, we recommend further cross-cultural studies on the issue of loot boxes to better understand where and why sociocultural differences may occur.

One notable finding is that in both datasets, at least some players reported spending at, or above parity, suggesting that at least some participants are reporting spending *more than they report they have as disposable income* each month on loot boxes. Critically, by definition, this suggests that for at least some players, loot boxes are potentially financially harmful. These findings also concord with research showing that a small proportion of players spend a large amount of money on loot boxes (e.g. [6]).

In at least some contexts, as participants spend a greater proportion of their disposable income on loot boxes, psychological distress increases and psychological well-being decreases. This replicates previous findings suggesting that loot boxes are associated with psychological distress (though the causal directionality of this association remains unknown), and extends work demonstrating an association between psychological distress and loot box spending [6,14,15].

Etchells *et al*.’s [16] and Xiao *et al*.’s [17] works offer vital methodological insight for future research. As might have been obvious all along, when considering psychological distress, the question is not necessarily whether individuals are spending more, but should be whether individuals are spending more *relative to what they can afford*. This subtle but important distinction helps in highlighting the players at risk from overspending and move the field towards better understanding where and when financial harms may be more or less likely. Notably, the measures employed by these studies for disposable income are relatively simplistic, and may be noisy in the way they relate to actual disposable income. However, when included in the analysis it is worth noting that for the Etchells *et al*.’s [16] dataset, the evidence for the presence of an effect of both estimated disposable income and loot box spending as a function of disposable income was 60 septillion times more likely than the null, suggesting that in the context of these data the self-report variable was contributing something meaningful to the model. It is possible that the variable might index how financially well-off individuals feel without necessarily being a highly accurate index of disposable income. We therefore suggest that future studies seek to employ validated scales or objective financial data to better understand this vital issue and improve precision in measurement of disposable income moving forwards. We also note that the current approach (i.e. the calculation of loot box spending as a ratio of disposable income) leads to a number of participants being excluded from the dataset (*n* = 107 or <4% in Etchells *et al*.’s [16] reanalysis, *n* = 450 or approximately 17% in Xiao *et al*.’s [17] reanalysis) because they did not report disposable income, or reported zero disposable income. Thus, our conclusions are necessarily limited to individuals who report more than zero disposable income. It is likely that individuals with no disposable income may display higher psychological distress than their peers with disposable income, and if included this may somewhat weaken the relationship between LS : DI and psychological distress because many also appeared not to purchase loot boxes. Nonetheless, among those individuals who have more than zero disposable income, the relationship between LS : DI and psychological distress appears to hold, at least in the Etchells *et al.* [16] dataset. These caveats underscore the need for validated disposable income scales.

We congratulate Etchells *et al.* [16] and Xiao *et al.* [17] for highlighting the importance of disposable income as a potentially valuable predictor in future investigations of in-game spending behaviour. It also offers novel insight for the field: at least some players report spending, on average, more than their reported disposable income each month on loot boxes, and unsurprisingly, at least in Etchells *et al*.’s data, these players are more likely to report being psychologically distressed. Whether this bears out with more validated measures of disposable income, only time and replication efforts will tell. Although methodological questions remain about differences between the use of the K-10 versus the K-6, and theoretical questions remain about whether sociocultural differences might be responsible for discrepancies in the field, one thing is unquestionably apparent from these data—some players (approximately 0.3% in Etchells *et al*.’s [16] data and 3.4% in Xiao *et al*.’s [17] data) report spending more than their entire reported disposable income on loot boxes. This concords with the work on conventional gambling which shows some gamblers bet more than they can afford to lose [32,33] and meets the quintessential definitions of financial harm.

## Data Availability

The data complete with analyses in JAMOVI are available at OSF [34].
